# Human periodontal ligament stem cells on calcium phosphate scaffold delivering platelet lysate to enhance bone regeneration†

**DOI:** 10.1039/c9ra08336g

**Published:** 2019-12-13

**Authors:** Zeqing Zhao, Jin Liu, Michael D. Weir, Ning Zhang, Li Zhang, Xianju Xie, Charles Zhang, Ke Zhang, Yuxing Bai, Hockin H. K. Xu

**Affiliations:** Department of Orthodontics, School of Stomatology, Capital Medical University Beijing China tuzizhangke@163.com; Department of Advanced Oral Sciences and Therapeutics, University of Maryland Dental School Baltimore MD 21201 USA; Key Laboratory of Shanxi Province for Craniofacial Precision Medicine Research, College of Stomatology, Xi'an Jiaotong University China; Member, Marlene and Stewart Greenebaum Cancer Center, University of Maryland School of Medicine Baltimore MD 21201 USA; Center for Stem Cell Biology & Regenerative Medicine, University of Maryland School of Medicine Baltimore MD 21201 USA

## Abstract

Human periodontal ligament stem cells (hPDLSCs) are promising for tissue engineering applications but have received relatively little attention. Human platelet lysate (HPL) contains a cocktail of growth factors. To date, there has been no report on hPDLSC seeding on scaffolds loaded with HPL. The objectives of this study were to develop a calcium phosphate cement (CPC)–chitosan scaffold loaded with HPL and investigate their effects on hPDLSC viability, osteogenic differentiation and bone mineral synthesis for the first time. hPDLSCs were harvested from extracted human teeth. Scaffolds were formed by mixing CPC powder with a chitosan solution containing HPL. Four groups were tested: CPC–chitosan + 0% HPL (control); CPC–chitosan + 2.66% HPL; CPC–chitosan + 5.31% HPL; CPC–chitosan + 10.63% HPL. Scanning electron microscopy, live/dead staining, CCK-8, qRT-PCR, alkaline phosphatase and bone minerals assay were applied for hPDLSCs on scaffolds. hPDLSCs attached well on CPC–chitosan scaffold. Adding 10.63% HPL into CPC increased cell proliferation and viability (*p* < 0.05). ALP gene expression of CPC–chitosan + 10.63% HPL was 7-fold that of 0% HPL at 14 days. Runx2, OSX and Coll1 of CPC–chitosan + 10.63% HPL was 2–3 folds those at 0% HPL (*p* < 0.05). ALP activity of CPC–chitosan + 10.63% HPL was 2-fold that at 0% HPL (*p* < 0.05). Bone minerals synthesized by hPDLSCs for CPC–chitosan + 10.63% HPL was 3-fold that at 0% HPL (*p* < 0.05). This study showed that CPC–chitosan scaffold was a promising carrier for HPL delivery, and HPL in CPC exerted excellent promoting effects on hPDLSCs for bone tissue engineering for the first time. The novel hPDLSC–CPC–chitosan–HPL construct has great potential for orthopedic, dental and maxillofacial regenerative applications.

## Abstract 2

Human periodontal ligament stem cells (hPDLSCs) are promising for tissue engineering applications but have received relatively little attention.

## Graphical abstract

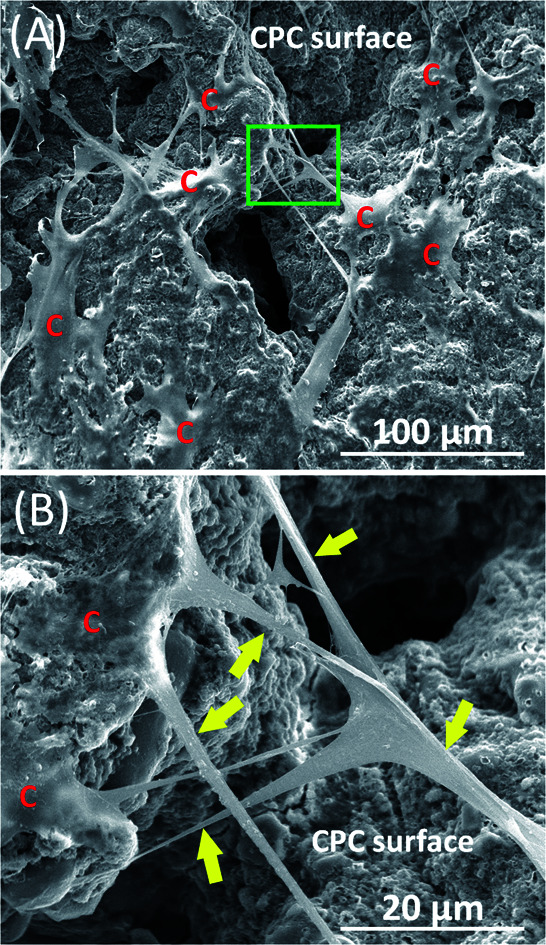

## Introduction

Due to congenital malformations, trauma, skeletal diseases and tumor resections,^1–3^ the need for bone repair and regeneration has been increasing. Bone tissue engineering uses scaffolds and cells to regenerate bone defects, and offers exciting potential to meet the need for bone repair and regeneration.^4^ Various types of scaffolds have been used in bone tissue engineering research, including metals, polymers, ceramics, calcium sulfates, and calcium phosphates (CaPs).^5^ Calcium phosphate cement (CPC) is a promising scaffold. CPC contains nanostructured minerals that mimic the bone extracellular matrix. The CPC mineral has similar chemical and crystallographic characteristics to the natural bone matrix minerals.^6,7^ The advantages of CPC include excellent biocompatibility, osteoconductivity, bioresorbability, injectability and the capability of self-setting to form a scaffold.^8,9^ In one formulation, the CPC powder consists of a mixture of tetracalcium phosphate (TTCP) and dicalcium phosphate anhydrous (DCPA).^5^ A CPC paste can be formed by mixing the CPC powder with a CPC liquid, leading to apatite precipitation as the end product.^5^

Combining cells with scaffolds is a potent strategy in tissue engineering.^10–13^ The seeded cells can differentiate into the osteogenic lineage and/or release bioactive molecules that induce osteogenesis to enhance the bone repair efficacy.^14^ Previous studies have shown positive results in stem cell delivery *via* scaffolds for bone regeneration.^15,16^ Human periodontal ligament stem cells (hPDLSCs) are a seed cell source that shows great potential for bone tissue engineering and especially dental and periodontal tissue regeneration, but yet have received relatively little attention.^17^ Besides the osteogenic lineage, hPDLSCs can also differentiate into several other cell lineages, including cementoblasts,^18,19^ chondrocytes,^18^ fibroblasts,^17^ and adipocytes.^20^ Therefore, hPDLSCs are a potent cell source for craniofacial and orthopedic tissue regeneration.

Human bone marrow mesenchymal stem cells (hBMSCs) are considered the gold-standard seed cells of bone tissue engineering research. However, hPDLSCs were shown to be more proliferative and clonogenic than hBMSCs.^20^ In addition, hBMSCs require an invasive procedure to acquire. In contrast, hPDLSCs can be harvested from the extracted third molar teeth, supernumerary teeth or the teeth extracted for orthodontic purpose, without the need for an extra surgery. Therefore, hPDLSCs are a relative easily accessible and low-cost source of stem cells, without the invasive procedures needed to harvest hBMSCs. hPDLSCs can be harvested from the extracted teeth for patients who need to have the teeth extracted for other medical reasons; for example, patients who need to have their wisdom teeth extracted, or orthodontic patients who need to have several premolars extracted. Therefore, the purpose is to avoid incurring an additional surgery for the patient. The hPDLSCs thus harvested can be frozen and used in the future as an autologous cell source to treat the same patient, thus avoiding immune rejection. In addition, besides differentiating into osteoblasts to form bone, hPDLSCs can also differentiate into lineages that form the periodontal tissues including cementum and periodontal fibers. In contrast, a literature search did not reveal any report that indicates that hBMSCs could differentiate into periodontal ligament fibers. Therefore, compared to hBMSCs, the hPDLSCs are a better cell source for the repair and regeneration of the periodontium. Hence, hPDLSCs are an exciting and promising alternative to the hBMSCs for alveolar bone repair and regeneration. However, hPDLSCs are a relatively new cell source for bone tissue engineering research, and to date, there has been no report on the seeding of hPDLSCs on CPC scaffold.

Recently, platelet derivatives have received attention in the field of bone tissue engineering. Human platelet lysate (HPL) is the product of the disruption of platelet membranes. HPL contains a cocktail of important growth factors, including platelet-derived growth factor (PDGF), fibroblast growth factor (FGF), vascular endothelial growth factor (VEGF), platelet-derived angiogenesis factor (PDAF), transforming growth factor (TGF), insulin-like growth factor (IGF), platelet factor 4 (PF-4) and platelet-derived epidermal growth factor (PDEGF).^21–23^ Bernardi *et al.* quantified the concentrations of the various growth factors in HPL.^24^ They reported that 1 mL HPL contained about 0.56 × 10^3^ pg VEGF, 25.16 × 10^3^ pg PDGF-AB, 4.78 × 10^3^ pg PDGF-AA, 5.06 × 10^3^ pg PDGF-BB, 53.04 × 10^3^ pg TGF-β1, and 0.085 × 10^3^ pg FGF-basic.^24^ Osteogenesis is a complex process which involves a variety of molecules,^25^ thus it is tempting to predict that applying all these molecules in HPL would have synergistic effects to enhance the bone regeneration. Indeed, a few studies indicated that HPL promoted the proliferation and osteodifferentiation of mesenchymal stem cells (MSCs), and hence HPL may represent a good therapeutic candidate for bone repair applications.^25,26^

However, a literature search revealed only one report that investigated the effect of HPL on hPDLSCs.^27^ In that study, HPL was added into the culture medium, without being delivered *via* a scaffold. The HPL benefited the matrix production by the hPDLSCs, the osteogenic gene expressions were upregulated, and the mineralization of hPDLSCs was increased.^27^ Therefore, combining hPDLSCs with HPL is indeed a promising approach. However, that previous did not test the use of a scaffold. For tissue engineering and bone regeneration, it would be beneficial to use a scaffold to deliver the HPL along with hPDLSCs. To date, there has been no report on the seeding of hPDLSCs on scaffolds loaded with HPL.

Several studies attempted to add growth factors into calcium phosphate cement to enhance osteogenesis, but CPC was brittle and mechanically weak.^28,29^ Our previous studies showed that adding a biopolymer, chitosan, into CPC produced a CPC–chitosan composite scaffold that was an effective delivery vehicle for drugs and biomolecules, and the scaffold had greater strength and fracture resistance than CPC control without chitosan.^30,31^

Accordingly, the objectives of present study were to develop a CPC–chitosan composite scaffold loaded with HPL, and to investigate their effects on the viability, osteogenic differentiation and bone matrix mineral synthesis of the seeded hPDLSCs for the first time. It was hypothesized that: (1) hPDLSCs would attach and grow well on the CPC–chitosan scaffold, hence the CPC–chitosan scaffold would be suitable to deliver hPDLSCs; (2) adding HPL into the CPC–chitosan scaffold would substantially increase the osteogenic gene expressions, alkaline phosphatase (ALP) activity, and bone mineral synthesis of the hPDLSCs, compared to those without HPL; (3) the osteogenesis efficacy of hPDLSCs on CPC–chitosan composite scaffold would be directly proportional to the HPL concentration in the scaffold.

## Materials and methods

### Fabrication of CPC scaffold

The CPC powder consisted of a mixture of TTCP and DCPA.^5^ TTCP was synthesized from a solid-state reaction between DCPA and calcium carbonate (CaCO_3_) (both from Baker Chemical, Phillipsburg, NJ, USA) in a furnace (Model 51333, Lindberg, Watertown, WI, USA) at 1500 °C for 6 hours. Then, the mixture was quenched to room temperature, ground in a ball mill and sieved to get TTCP powder with a median particle size of 5 μm. DCPA was ground for 24 hours to obtain particles with a median sizes of 1 μm. TTCP and DCPA were mixed at 1 : 3 molar ratio to obtain the CPC powder.^32,33^ CPC powder, chitosan lactate powder (Technical grade, VANSON, Redmond, WA, USA; referred to as chitosan), and specimen molds were sterilized in an ethylene oxide sterilizer (Andersen, Haw River, NC, USA) for 24 hours according to the manufacturer, and degassed for 7 days prior to making the specimens. Human platelet lysate (HPL) was obtained in the form of a liquid (Zenbio, NC, USA). According to the manufacturer, the HPL was human platelet-derivative obtained from pooled platelet-rich plasma by one or more cycles of freezing and thawing to mechanically disrupt the platelet-membranes. Thus, the HPL contained the entire intra-cellular contents released from the platelets.

The as-received HPL solution was added into sterile distilled water at various concentrations to make several different solutions. CPC liquid was made by dissolving the chitosan powder into each of the aforementioned solutions, at a chitosan/(chitosan + solution) mass fraction of 15%.^31^ CPC paste was formed by mixing the CPC powder with each CPC liquid at a powder to liquid ratio of 3 : 1 by mass.^31^ Calculations of the all the individual components of the composite scaffolds yielded the final mass fraction of HPL in each CPC paste. The HPL mass fraction in the final CPC = the as-received HPL mass/(CPC powder + the as-received HPL + distilled water + chitosan powder) = 0%, 2.66%, 5.31% and 10.63%, respectively (all mass fractions). This yielded four groups of CPC paste.

Each CPC paste was filled into a disk mold of 10 mm in diameter and 1 mm in thickness. Each CPC sample was formed by using 100 mg of the mixed CPC paste. The disks were placed in a humidor incubator for 24 hours at 37 °C. Four groups of specimens were thus fabricated. The components of all the groups are listed in Table 1.

**Table 1:** Component of one CPC sample

Group name	CPC powder (mg)	Chitosan powder (mg)	HPL (mg)	Distilled water (mg)
0% HPL group (control)	75	3.75	0	21.25
2.66% HPL group	75	3.75	2.66	18.59
5.31% HPL group	75	3.75	5.31	15.94
10.63% HPL group	75	3.75	10.63	10.62

### hPDLSC culture and seeding

Human premolars extracted for orthodontic purposes were collected with informed consent. All experiments were performed in accordance with the guidelines of the National Institutes of Health (NIH), and the experiments were approved by the Institutional Review Board (IRB) Committee at the University of Maryland Baltimore (approval ID: HP-00079029). The periodontal ligaments fragments were digested to cell suspension in a solution of 3 mg mL^−1^ collagenase type I (Millipore, St. Louis, MO, USA) and 4 mg mL^−1^ of dispase (Sigma-Aldrich, St. Louis, MO, USA). The cell suspension was seeded in culture dishes with a growth medium consisting of DMEM (Gibco, Gaithersburg, MD, USA), which was supplemented with 10% FBS (HyClone, Logan, UT, USA), 2 mM l-glutamine (Gibco), 100 U mL^−1^ penicillin and 100 mg mL^−1^ streptomycin (Gibco). The samples were incubated at 37 °C in 5% carbon dioxide. Colonies formed by the cells were detached using 0.25% trypsin–EDTA, and seeded in 24-well plate. When the confluency reached 70% to 80%, the cells were detached and transferred to 50 mm culture dishes for expansion. When the confluency again reached 70% to 80%, the cells were passaged and transferred to 100 mm dishes for further expansion. The culture medium was changed every other day. The cells were detached and passaged when the confluency reached 70% to 80%.

The hPDLSCs of passage 3–5 were used in subsequent experiments. A seeding density of 5 × 10^4^ cells diluted in 0.5 mL of growth medium was seeded drop-wise onto each CPC–chitosan scaffold disk, which was placed in a 24-well plate. On the second day, the medium was changed to an osteogenic medium which consisted of DMEM and supplemented with 10% FBS, 2 mM l-glutamine, 100 U mL^−1^ penicillin and 100 mg mL^−1^ streptomycin, 100 nM dexamethasone, 10 mM β-glycerophosphate, 0.05 mM ascorbic acid, and 10 nM 1α,25-dihydroxyvitamin (Millipore).^34^

### Scanning electron microscopy of hPDLSCs on CPC

The cell-scaffold constructs were examined under scanning electron microscopy (SEM, Quanta 200, FEI, Hillsboro, OR, USA). Samples were fixed with 1% glutaraldehyde (Millipore), subjected to graded alcohol dehydrations (30–100%), and rinsed with hexamethyldisilazane (Millipore), and examined with SEM.

### Live/dead staining and CCK-8 assay

After culturing for 1, 7 and 14 days, a live/dead staining kit (Molecular Probes, Eugene, OR, USA) was used to test the viability of the cells seeded on CPC. The cell-seeded disks were washed with PBS and incubated with 4 mM ethidium homodimer-1 and 2 mM calcein-AM in PBS for 20 min. The disks were then viewed by using epifluorescence microscopy (Eclipse TE2000-S, Nikon, Melville, NY, USA). The live cell density was measured as *D* = the number of live cells in the image/the image area.^32^ The percentage of live cells was measured as *P* = the number of live cells/(the number of live cells + the number of dead cells) in the same image.^32^ For each specimen, three fields of view were randomly chosen and photographed, yielding 15 images per group (*n* = 5) at each time point for each of the five CPC–chitosan–HPL scaffold groups.

Separate specimens were cultured and tested using a cell counting kit (CCK-8, Dojindo, Tokyo, Japan) to quantify cell proliferation at 1, 4, 7, 10 and 14 days. CPC disks were immersed in the medium containing 10% CCK-8, and incubated in dark for 2 hours. Then the medium was transferred to a 96-well plate. The cell proliferation was examined *via* the absorbance at an optical density of 450 nm using a microplate reader (SpectraMax M5, Molecular Devices, Sunnyvale, CA, USA). Six replicates in each group were used (*n* = 6).

### Quantitative real time-polymerase chain reaction (qRT-PCR)

A qRT-PCR (7900HT, Applied Biosystems, Foster City, CA, USA) method was used to measure the osteogenic differentiation of hPDLSCs on CPC scaffolds. After 1, 7 and 14 days of culture, the total cellular RNA of hPDLSCs on the scaffold was extracted with TRIzol reagent (Invitrogen, Grand Island, NY, USA) and reverse-transcribed into cDNA using a high-capacity cDNA reverse transcription kit (Applied Biosystems) in a thermal cycler (GenAmp PCR 2720, Applied Biosystems). RT^2^ SYBR® Green qPCR Mastermix (Qiagen, Germantown, MD, USA) was used to quantify the expression of the targeted genes for alkaline phosphatase (ALP), Runt-related transcription factor (Runx2), osterix (OSX), collagen type I (Coll1), and glyceraldehyde 3-phosphate dehydrogenase (GAPDH). The relative expression level for each target gene was evaluated using the 2^−ΔΔ*C*_*t*_^ method. The *C*_*t*_ values of the genes were normalized by the *C*_*t*_ values of the human housekeeping gene GAPDH. The *C*_*t*_ value of hPDLSCs in control group at 1 day was used as the calibrator.

### ALP activity assay

After culturing in the osteogenic medium for 7 and 14 days, cells on the CPC scaffolds were detached with 0.25% pf trypsin–EDTA. Then cells were lysed in a 0.2% Triton X-100 (Millipore Sigma) solution for 20 min. The ALP activity in lysates was quantified using Alkaline Phosphatase Assay kit (QuantiChrom, BioAssay Systems, Hayward, CA, USA). SpectraMax M5 microplate reader (Molecular Devices) was used to examine the ALP activity at an optical density of 450 nm. The ALP activity was normalized to protein concentration for each sample, using a Protein Assay Kit (Pierce BCA, Thermo Scientific, Rockford, IL, USA).

### Mineral synthesis by the hPDLSCs

At 1, 7 and 14 days, the hPDLSC–CPC disks were fixed with 10% formaldehyde for 30 minutes and stained with Alizarin Red S (ARS, Millipore, Billerica, MA) for 30 minutes (*n* = 6). The ARS stained the mineral deposits synthesized by the cells into a red color. After staining, the ARS solution was removed, and the disks were washed gently with PBS to remove any loose ARS. Then the specimens were photographed. The CPC disks were soaked in 10% cetylpyridinium chloride (Millipore) for 1 hour to extract the stained deposit.^35^ The ARS concentration was measured at optical density of 652 nm using the microplate reader (SpectraMax M5). Blank CPC scaffolds with the same treatments, but without cell seeding, were also measured. The value of blank CPC scaffolds was subtracted from the value of the cell-seeded scaffolds to calculate the mineral concentration synthesized by the cells.^36^ The concentration of control group at 1 day was used as the calibrator.

### Statistical analyses

Statistical analyses were performed using Statistical Package for the Social Sciences (SPSS 19.0, Chicago, IL, USA). All data were presented as the mean value ± standard deviation (SD). Significant (*p* = 0.05) effects of the variables was detected using two-way ANOVA, followed by post hoc LSD (least significant difference) tests.

## Results

**Fig. 1:**
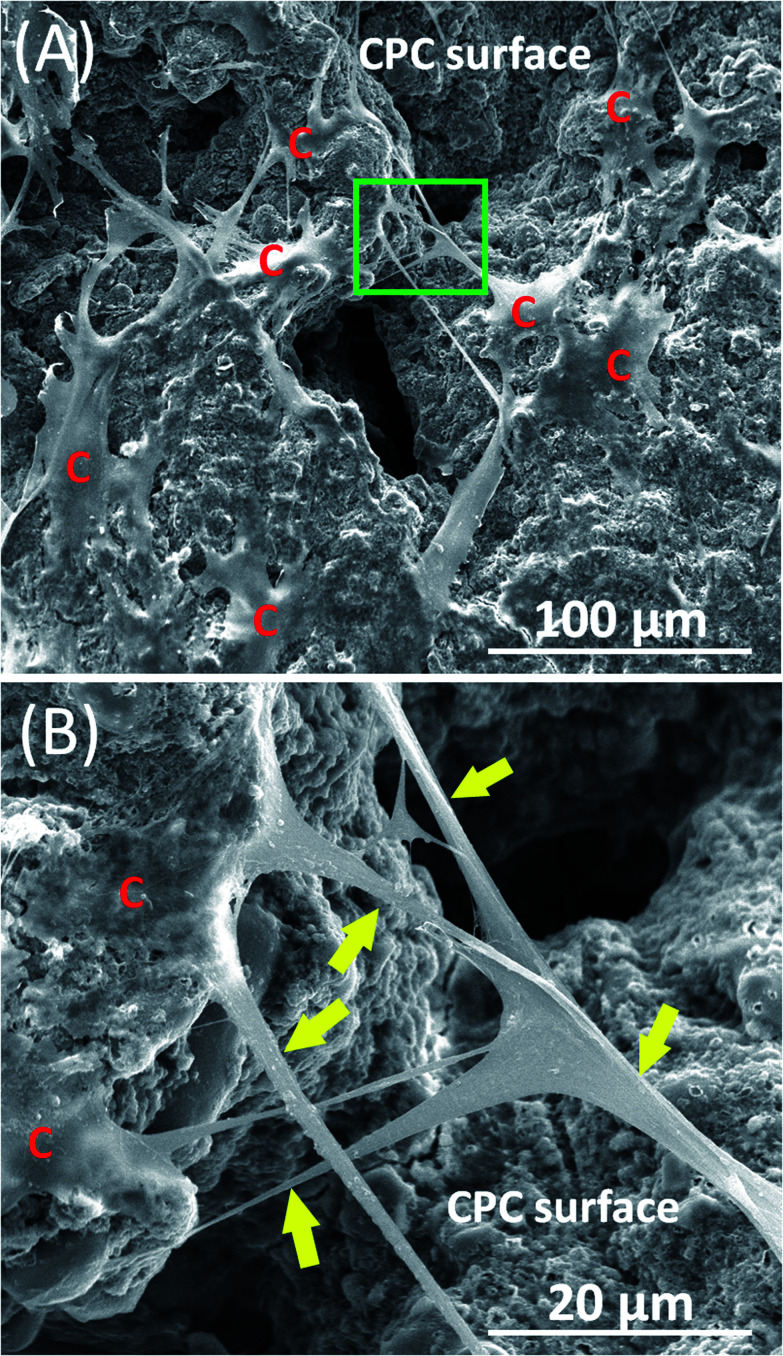
Representative SEM images of hPDLSCs on CPC scaffolds for 14 days. (A) shows hPDLSCs attaching on CPC scaffold. ‘‘C’’ stands for hPDLSCs. (B) is the higher magnification image of green dotted frame in (A). (B) shows that cell extensions (yellow arrows) anchored to the CPC scaffold.

Fig. 1 shows typical SEM images of hPDLSCs (referred to as “C”) attaching to a CPC–chitosan scaffold after culturing for 14 days. Fig. 1A shows that hPDLSCs attached very well to the CPC–chitosan scaffold. Fig. 1B is an higher magnification image of the green dotted frame in Fig. 1A. Fig. 1B shows that hPDLSCs developed cytoplasmic extensions (yellow arrows) and the cell extensions attached to the CPC scaffold. This indicated that CPC–chitosan scaffold had good biocompatibility for the hPDLSCs.

**Fig. 2:**
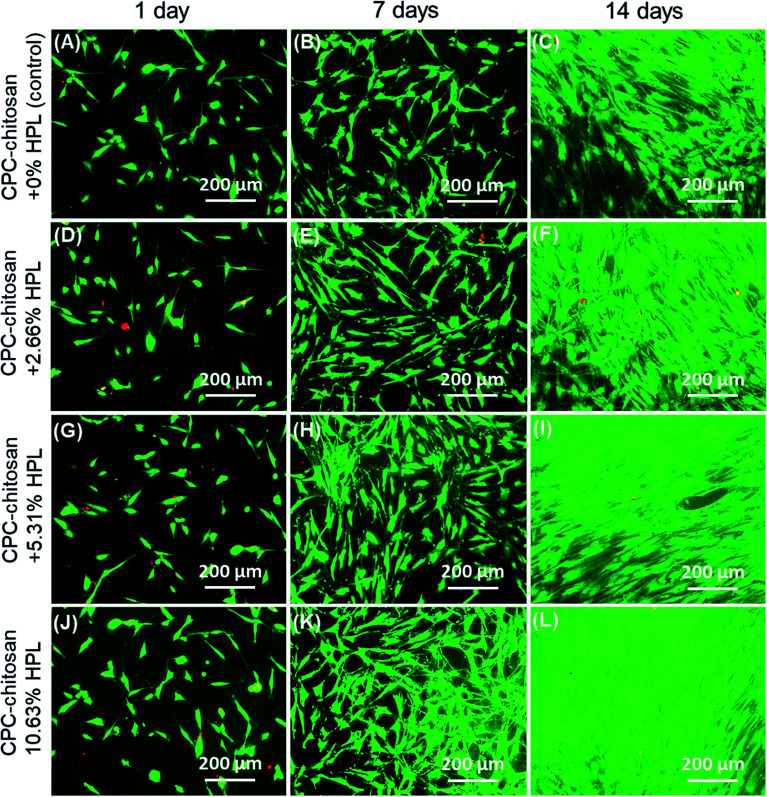
(A)–(L) are the representative live/dead staining images of hPDLSCs on CPC scaffolds for 1, 7 and 14 days for: CPC–chitosan + 0% HPL (control); CPC–chitosan + 2.66% HPL; CPC–chitosan + 5.31% HPL; CPC–chitosan + 10.63% HP. The number of live cells (stained green) increased with time. Live cells were numerous while the dead cells (stained red) were few. Cells of all groups appeared to be well attached to CPC scaffold.

Fig. 2A–L show representative live/dead images of hPDLSCs on CPC scaffolds at 1, 7 and 14 days, respectively. The hPDLSCs grew well on CPC scaffolds. There were many more cells at 14 days than 1 day due to cell proliferation on CPC. The live cells (stained green) were numerous, while the dead cells (stained red) were few. Cells of all groups appeared to be well attached to the CPC–chitosan surface. This indicates that the CPC–chitosan scaffolds with various HPL concentrations had good biocompatibility for the hPDLSCs.

**Fig. 3:**
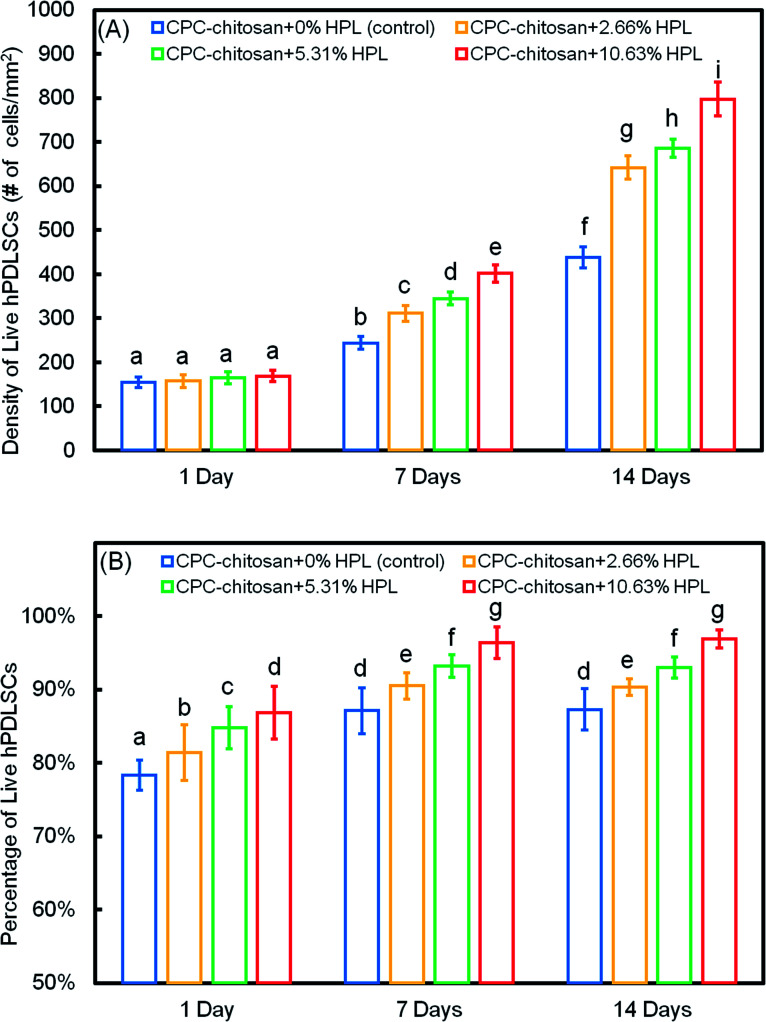
hPDLSC viability on CPC scaffolds (mean ± sd; *n* = 5). (A) The live cell density of all groups increased with time due to proliferation. (B) The percentage of live cells of all groups was about 70% to 90%. Values indicated by dissimilar letters are significantly different from each other (*p* < 0.05).

The quantification in Fig. 3A showed that the live cell density of all groups increased with time due to proliferation. At 7 and 14 days, the live cell densities of the HPL groups were significantly higher than that of the control group (*p* < 0.05). In addition, for the HPL groups, the live cell density increased with HPL concentration. In Fig. 3B, the percentages of live cells of all groups ranged from about 70% to 90%. The percentages of live cells at 7 and 14 days were higher than that at 1 day. At 7 and 14 days, the CPC–chitosan + 10.63% HPL group had the highest live cell percentage (96.4% and 96.9%), while those of the control group were 80.9% and 83.3%. The results revealed that, compared to the control group, the cell proliferation was increased with the addition of HPL.

**Fig. 4:**
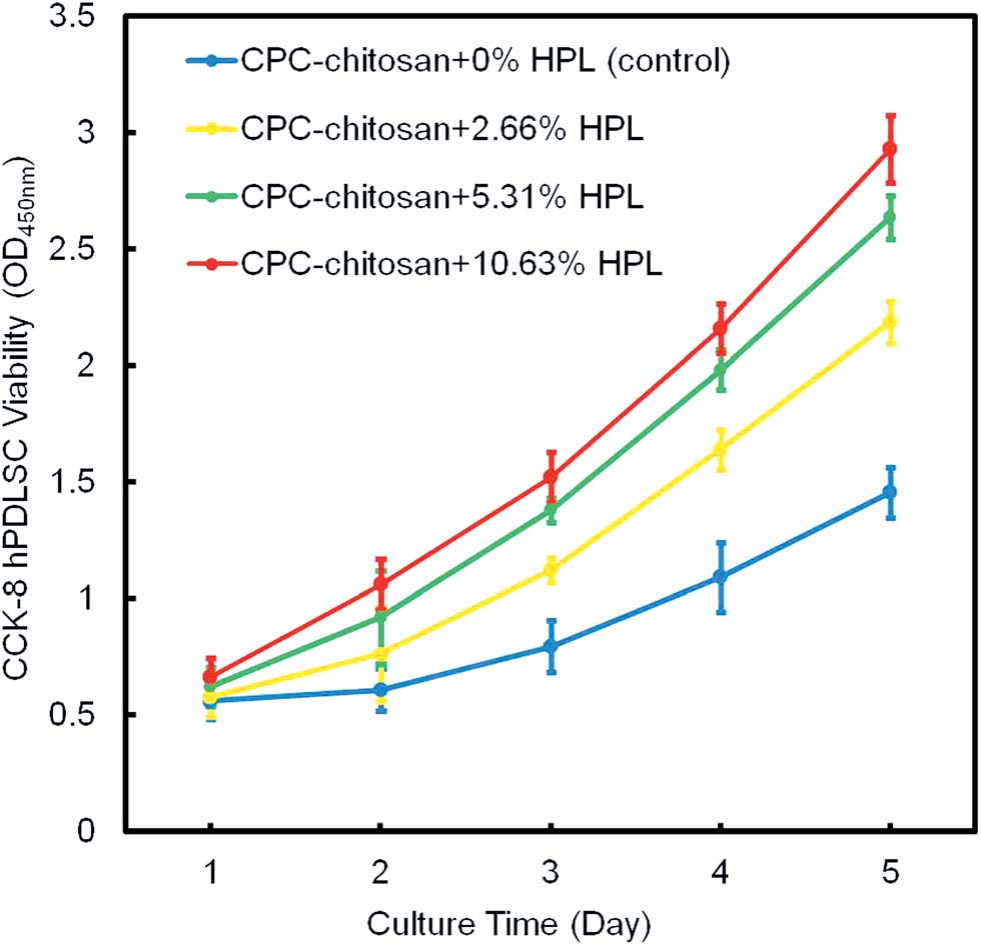
The hPDLSC proliferation on CPC scaffolds *via* CCK-8 assay (mean ± sd; *n* = 6). CPC–chitosan + 10.63% HPL group had the greatest cell proliferation rate.

Fig. 4 shows the result of the CCK-8 assay. For all groups, the cells proliferated well from 1 day to 14 days. Compared to the control group, the proliferation of the HPL groups was faster (*p* < 0.05). The CPC–chitosan + 10.63% HPL group demonstrated the highest cell proliferation which was increased by 4 folds from 1 day to 14 days, while the control group was increased by only 2 folds.

**Fig. 5:**
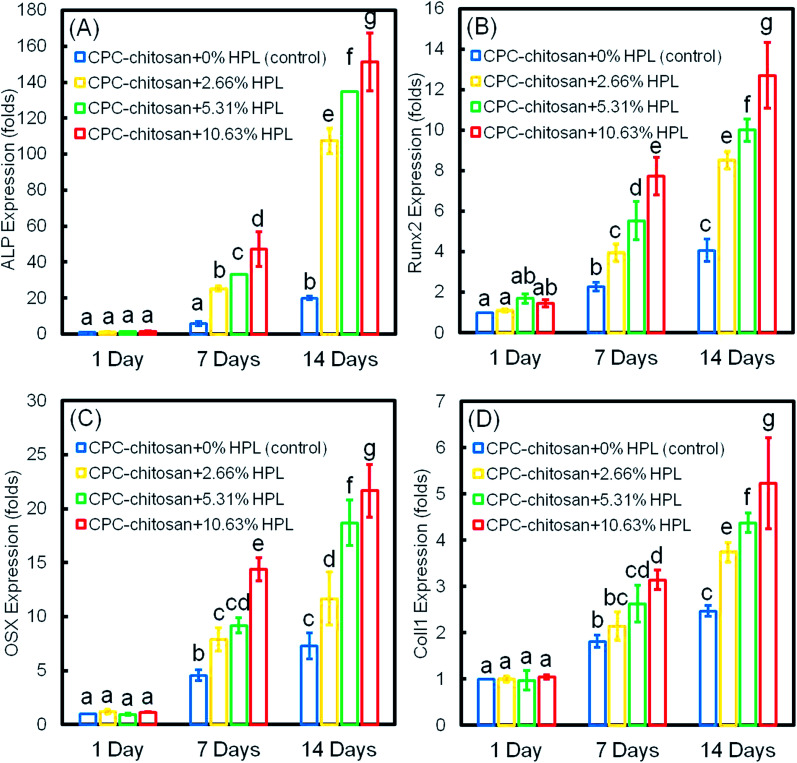
Osteogenic differentiation of hPDLSCs on CPC: (A) ALP, (B) Runx2, (C) OSX, and (D) Coll1 gene expressions (mean ± sd; *n* = 6). The expression of all four genes was elevated with culturing time, and they peaked at 14 days. The incorporation of HPL upregulated the osteogenic differentiation, and hPDLSCs in CPC–chitosan + 10.63% HPL group had the highest expression of osteogenic genes among all groups (*p* < 0.05). Values indicated by dissimilar letters are significantly different from each other (*p* < 0.05).

The osteogenic differentiation results of hPDLSCs seeded on CPC are plotted in Fig. 5: (A) ALP, (B) Runx2, (C) OSX, and (D) Coll1 gene expressions. The expression of all four genes was elevated with culture time and peaked at 14 days. The incorporation of HPL into CPC scaffolds upregulated the osteogenic gene expression at 7 and 14 days. CPC–chitosan + 10.63% HPL group had the highest expression of osteogenic genes among all the groups (*p* < 0.05). The ALP gene expression of this group was about 7-fold that of CPC control group without HPL at 14 days. For Runx2, OSX and Coll1, the gene expression of CPC–chitosan + 10.63% HPL group was about 2-fold to 3-fold that of the CPC control group with 0% HPL.

**Fig. 6:**
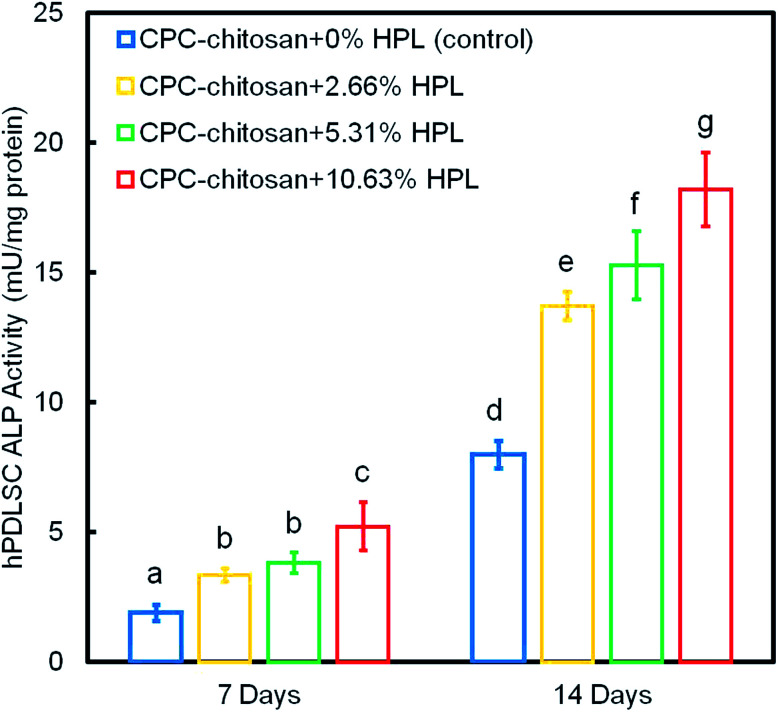
ALP protein activity of hPDLSCs on CPC scaffolds at 7 and 14 days (mean ± sd; *n* = 6). The ALP activity of all groups was elevated with time. The groups containing HPL reached a higher ALP activity than that at 0% HPL (*p* < 0.05). The ALP activity of hPDLSCs in the CPC–chitosan + 10.63% HPL group was the highest among all groups. Values indicated by dissimilar letters are significantly different from each other (*p* < 0.05).

Fig. 6 shows the ALP protein activity of hPDLSCs on CPC scaffolds. The ALP activity of all groups was elevated with increasing culture time. The addition of HPL increased the ALP activity of the hPDLSCs (*p* < 0.05). In addition, the concentration of HPL is positively correlated with the ALP activity. Among all four groups, the ALP activity of CPC–chitosan + 10.63% HPL group was the highest, which was more than 2-fold that of CPC control group without HPL at 14 days.

**Fig. 7:**
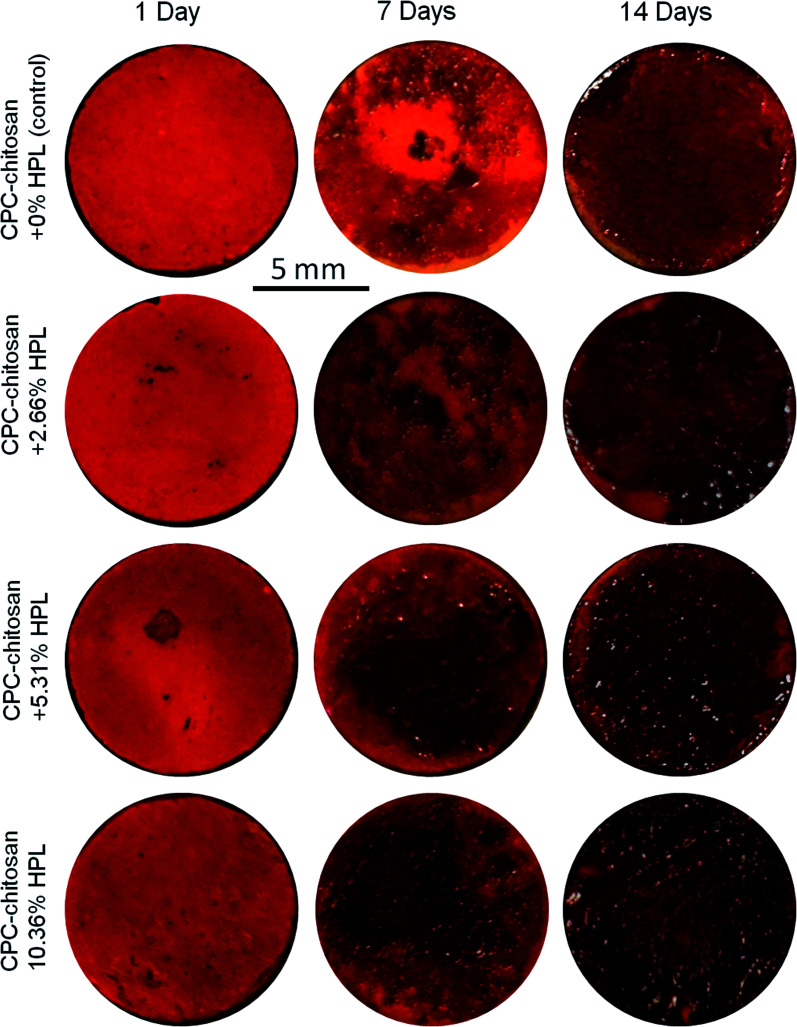
Representative ARS staining images of bone mineral synthesis by hPDLSCs on CPC. For all four groups, the color of CPC became a darker red from 1 day to 14 days. The red staining of groups with HPL was deeper and denser than that at 0% HPL.

Representative ARS staining images of mineral synthesis by hPDLSCs on CPC scaffolds are shown in Fig. 7. For all four groups, the mineral staining became darker from 1 day to 14 days due to the accumulation of mineral deposits made by the hPDLSCs. The red staining for the HPL groups was deeper and denser that without HPL at 7 and 14 days, as the mineralization was enhanced by incorporating HPL into CPC. The staining became a darker red with the increase of HPL concentration.

**Fig. 8:**
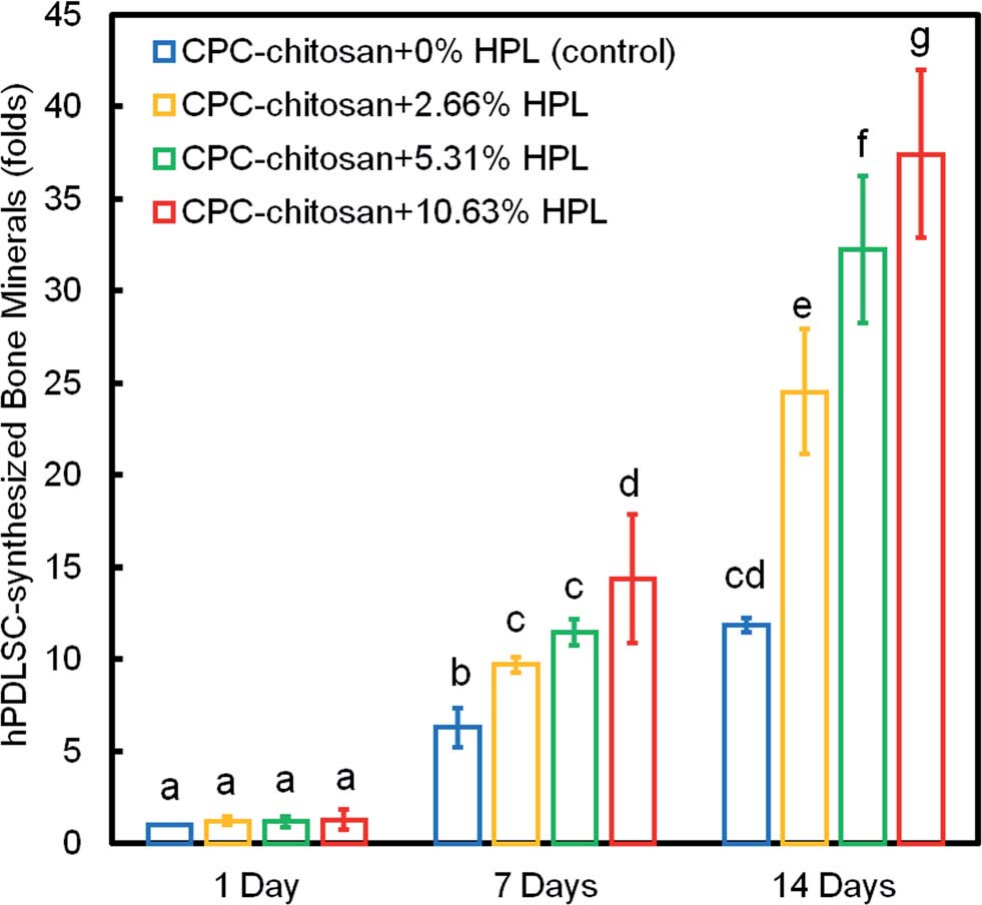
hPDLSC synthesis of bone minerals (mean ± sd; *n* = 6). The cell-synthesized mineral amount significantly increased from 1 to 14 days (*p* < 0.05). The HPL groups accumulated more minerals than that at 0% HPL (*p* < 0.05). hPDLSCs in the CPC–chitosan + 10.63% HPL group synthesized the most calcified deposit, which was about 3-fold that at 0% HPL. Values indicated by dissimilar letters are significantly different from each other (*p* < 0.05).

Fig. 8 shows the quantification of mineral synthesis by the hPDLSCs. The cell-synthesized mineral amount significantly increased from 1 day to 14 days (*p* < 0.05). The HPL groups accumulated more minerals than the control group with 0% HPL (*p* < 0.05). In particular, the hPDLSCs in CPC–chitosan + 10.63% HPL group synthesized the greatest amount of calcified deposits, which was about 3-fold that at 0% HPL.

**Fig. 9:**
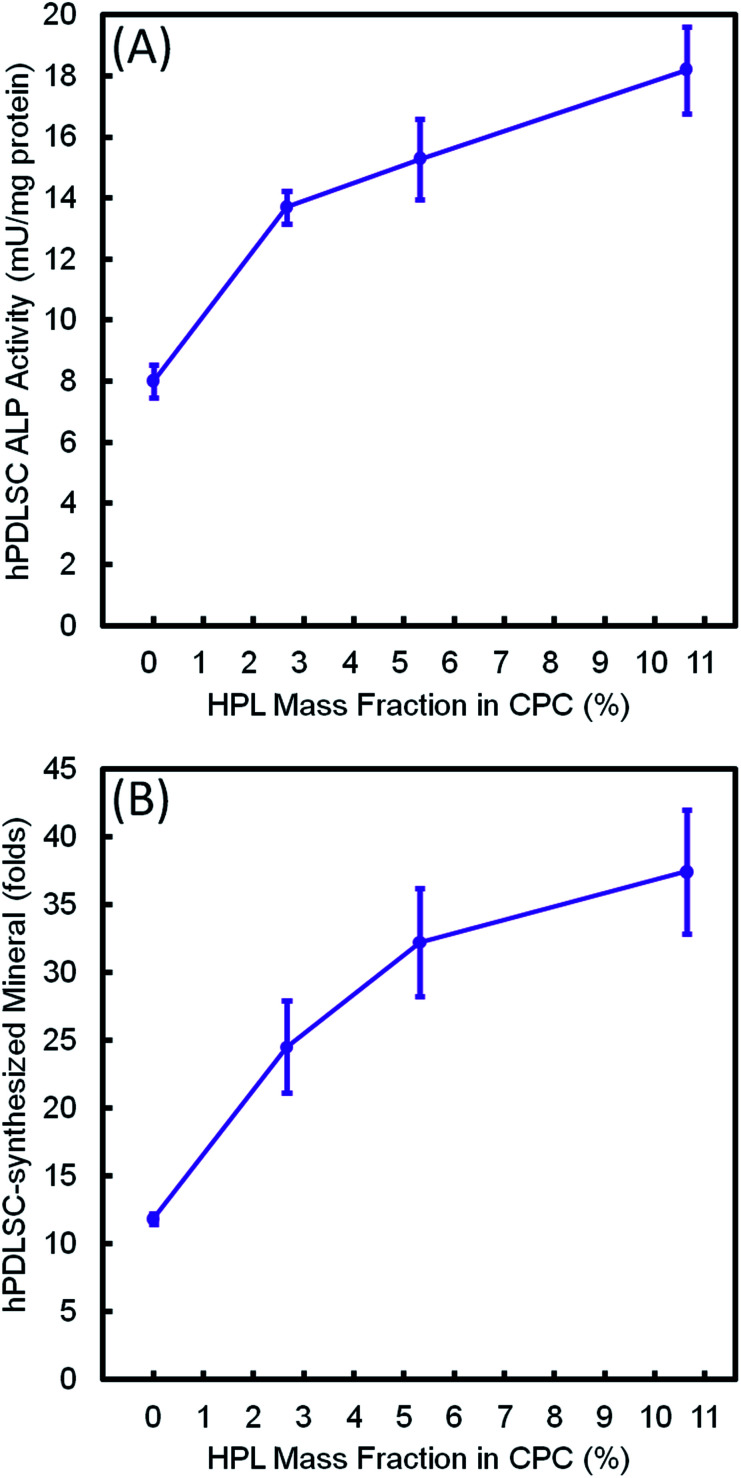
Effect of HPL mass fraction in CPC–chitosan scaffold on: (A) ALP activity, and (B) bone matrix mineral synthesis of hPDLSCs at 14 days (mean ± sd; *n* = 6). The incorporation of 10.65% HPL in CPC–chitosan increased the ALP activity and bone mineral synthesis by 2–3 folds. A directly proportional relationship was established between HPL mass fraction in CPC scaffold and ALP activity and bone mineral synthesis of hPDLSCs.

Fig. 9 plots the 14 day results for (A) ALP activity, and (B) bone matrix mineral synthesis of the hPDLSCs as a function of HPL mass fraction in CPC–chitosan scaffolds. The results showed that the CPC–chitosan scaffold without HPL also supported the ALP activity and bone matrix mineral synthesis of the hPDLSCs. However, incorporating 10.65% HPL in CPC increased the ALP activity and bone mineral synthesis by 2–3 folds. The ALP activity and bone mineral synthesis of hPDLSCs on the scaffold were directly proportional to the HPL mass fraction in CPC scaffold.

## Discussion

The present study represents the first report on the seeding and delivery of hPDLSCs on CPC–chitosan scaffolds loaded with HPL to enhance bone regeneration. The hypotheses were proven that the hPDLSCs harvested from the extracted human teeth attached and proliferated well on CPC–chitosan scaffold, demonstrating the CPC–chitosan scaffold as feasible carrier to deliver hPDLSCs. In addition, the incorporation of HPL into the CPC–chitosan scaffold greatly increased the osteogenic gene expressions, ALP activity, and bone mineral synthesis of hPDLSCs. Furthermore, within a range of the tested HPL concentration, the osteogenesis efficacy of hPDLSCs including ALP activity and bone mineral synthesis were shown for the first time to be directly proportional to the HPL concentration in the CPC–chitosan scaffold. Therefore, the novel hPDLSC–CPC–chitosan–HPL construct is promising to promote the bone repair and regeneration efficacy.

In the present study, hPDLSCs were harvested from the extracted human teeth and used as seed cells to combine with the CPC scaffold. hPDLSCs can differentiate into multiple types of cells, such as osteoblasts, cementoblast-like cells, and collagen-forming cells under the right culture conditions.^20,37^ It was demonstrated that hPDLSCs formed a cementum-like tissue with condensed collagen fibers, which consisted of the periodontal ligament.^38^ Thus, the transplantation of hPDLSCs was suggested to be a promising alternative cell source to the gold-standard hBMSCs, and hold the promise as a therapeutic approach for the reconstruction of alveolar bone tissues and the periodontal complex.^38^

CPC scaffolds are excellent candidates for orthopedic surgical applications due to their excellent biocompatibility, osteoconductivity, degradability, injectability and moldability. However, their poor strength and brittleness have limited their use to primarily low stress-bearing applications.^30,39^ The diametral tensile strength of the protein-containing CPC was relatively low, ranging from 3.5 to 6.4 MPa.^40^ For the above reasons, a biopolymer chitosan was incorporated into CPC to improve the load-bearing capability and toughness of CPC. Previous studies demonstrated that the flexural strength of the CPC–chitosan composite scaffold was 19.8 MPa, which was more than 2 folds of the 8 MPa for the CPC control without chitosan.^30,41^ The strength of CPC–chitosan composite scaffold exceeded the flexural strength of sintered porous hydroxyapatite (2–11 MPa) and the tensile strength of cancellous bone (3.5 MPa), thus rendering the CPC–chitosan composite scaffold promising for a wide range of orthopedic, dental and craniofacial applications.^30,41^

Furthermore, the CPC–chitosan scaffold has potential to serve as a delivery vehicle for drugs and growth factors to promote bone regeneration. In our previous study, CPC–chitosan composite scaffold successfully delivered human bone morphogenic protein-2 and significantly enhanced the osteogenic differentiation of the cells.^30^ This is consistent with several other studies that showed that chitosan could help stabilize and maintain the bioactivity of growth factors to modulate stem cell differentiation.^42^ In addition, another study showed that CPC–chitosan scaffold was an effective delivery system with controlled release of metformin to promote osteogenic differentiation.^31^ Therefore, the CPC–chitosan composite scaffold possessed good mechanical properties and drug/growth factor delivering capability, making it a promising scaffold to deliver HPL for bone regeneration.

Previous studies^43,44^ added HPL to hyaluronic acid microparticles and then mixed with CPC to form a system for HPL delivery for bone regeneration. However, the incorporation of hyaluronic acid microparticles reduced the mechanical properties of the CPC.^43^ In contrast, in the present study, HPL was added into a chitosan solution. Instead of decreasing the scaffold strength, and the incorporation of chitosan + HPL could actually increase the mechanical property of CPC, compared to that without chitosan. Another study^26^ fabricated HPL-coated hydroxyapatite/β-tricalcium phosphate scaffolds by immediately immersing the scaffolds into a HPL solution. However, this strategy only allowed an external coating of HPL on the biomaterial, without providing a sustained release of HPL from the interior of the scaffold. In the present study, the HPL was mixed into the CPC–chitosan composite paste, and the HPL was distributed throughout the entire scaffold volume. This enabled a higher loading of HPL than that on the scaffold surface only, enabled a sustained release of HPL, and provided a tailored control of the dose of HPL in the scaffold. In the present study, HPL was proven to have dual effects on hPDLSCs: (1) HPL substantially enhanced the proliferation of hPDLSCs on CPC scaffold for the first time; (2) HPL highly promoted the osteogenic differentiation of hPDLSCs on CPC scaffold, generating bone mineral that was 3 folds that without HPL. Furthermore, these results also indicate that the CPC–chitosan scaffold was a effective carrier to deliver HPL, and that the CPC–chitosan–HPL construct was capable to deliver hPDLSCs for bone tissue engineering.

As the product of the disruption of platelet membranes, HPL constitutes a rich natural source of growth factors that can promote the essential stages of tissue repair.^44^ HPL contains a broad spectrum of growth factors that have been shown to participate in the advancement of MSC proliferation, including PDGF, TGF, FGF, IGF and VEGF, with most of them being stable for up to 2 weeks.^45^ In the present study, according to the results of live/dead staining and CCK-8, the HPL groups had significantly higher density and percentage of live cells than control group. In addition, the effect was enhanced with the increase of HPL concentration in CPC from 2.66% to 10.63% HPL. These results were consistent with a previous study^46^ that showed that a significant enhancement in human dental pulp stem cells (hDPSCs) proliferation was observed when using 1% and 5% HPL-containing culture media. The 5% HPL group had a higher cell proliferation rate. Therefore, HPL could increase the cell proliferation, and this function was dose-dependent.

Among all the growth factors contained in the HPL, PDGF, IGF, FGF and TGF are proven to induce the osteogenic differentiation of MSCs.^47^ PDGF activates BMP-Smad1/5/8 signaling and promote the MSCs to differentiate into osteoblasts *via* the BMP-Smad1/5/8-Runx2/Osterix pathway.^48^ IGF can stimulate bone formation by up-regulating type I collagen transcription and decreasing the synthesis of collagenase 3 or matrix metalloproteinase 13, a collagen-degrading protease.^49^ In addition, IGF could support osteoblastogenesis by stabilizing β-catenin, a signaling molecule used by the Wnt canonical signaling pathway, which is essential for osteoblastogenesis.^49^ Further more, FGF plays a key role in the osteogenesis by activating the Runx2 and influencing the regulation of bone formation.^50^ Moreover, FGF also contributed in the positive regulation of the bone growth and the anabolic function of osteoblasts.^48^ TGF induces early differentiation of osteoprogenitor cells by activating receptor-regulated Smads.^51^ TGF also initiates mitogen-activated protein kinase (MAPK) signaling cascade, which regulate the expression of collagen I and osteocalcin.^51^ Besides, TGF increases Runx2 expression during the early differentiation of osteoblasts.^51^

ALP, Runx2, OSX and Coll1 are all important osteogenic differentiation markers of hPDLSCs.^52–54^ In the present study, the results of qRT-PCR indicated that the expression of all four osteogenic genes was up-regulated for hPDLSCs in the groups with HPL. The ALP activity of groups with HPL was higher than that of control group. Moreover, the ARS staining confirmed that the HPL incorporation in CPC–chitosan strongly enhanced the cell mineralization (*p* < 0.05). Furthermore, the results of qRT-PCR, ALP activity assay and ARS staining indicated that the effect of HPL on cell differentiation varied with the dosage of HPL. Increasing the concentration of HPL in CPC from 2.66% to 10.63% greatly increased the osteogenic differentiation and mineral synthesis by the hPDLSCs. Among all the tested groups, the CPC–chitosan + 10.63% HPL scaffold yielded the best osteogenic differentiation and the greatest amount of mineral synthesis by the hPDLSCs. These results are consistent with a previous study^55^ that found that adding 5% HPL into the culture medium induced the osteogenic differentiation of hDPSCs in a shorter period of time with an increase of ALP activity as compared to the group with 1% HPL. Another study^46^ showed that 5% HPL in the culture medium resulted in the highest ALP activity for hDPSCs among all concentrations tested (1%, 5%, and 10%). However, these previous studies simply added the HPL into the culture medium, without using a scaffold. The present study represents the first study on incorporating HPL into CPC–chitosan scaffold and showing the substantial enhancements of cell proliferation, osteogenic differentiation and bone mineral synthesis *via* the novel hPDLSC–CPC–chitosan–HPL construct.

The results of the present study indicate that the various and controlled concentrations of HPL growth factors delivered *via* a bioactive scaffold caused different and tailored effects on the proliferation and osteogenic differentiation of stem cells, and that such promoting effects were dose-dependent. This study showed that the CPC–chitosan + 10.63% HPL scaffold resulted in the best hPDLSC proliferation, osteogenic differentiation and bone matrix mineralization. Further studies are needed to investigate the optimal dose of HPL in CPC–chitosan composite scaffold in an animal model, and to establish the relationship between HPL concentration *vs.* new bone formation and new blood vessel density in the defects *in vivo*. The present study represents the first report on harvesting and seeding hPDLSCs on CPC–chitosan scaffold, where the cells were attached to the scaffold surface. Further study is needed to seed the hPDLSCs into the interior of the scaffold, for example, *via* cell-encapsulating hydrogel microbeads or fibers distributed inside the CPC scaffold.^56,57^ The hydrogel microbeads and fibers could protect the cells during the CPC paste mixing and CPC setting reaction. Then the microbeads and fibers would degrade in a few days to release the cells throughout the CPC scaffold volume.^56,57^ Further study is needed to encapsulate hPDLSCs inside hydrogel in CPC to form a three-dimensional (3D) cell–CPC scaffold, as well as to investigate cell-encapsulating scaffolds using 3D printing techniques.

## Conclusions

This study represents the first report on a novel hPDLSC–CPC–chitosan–HPL construct for bone tissue engineering, demonstrating substantial increases in hPDLSC proliferation rate, osteogenic differentiation and bone mineral synthesis. hPDLSCs were harvested from the extracted human teeth. hPDLSCs attached and proliferated well on CPC–chitosan scaffold. HPL delivery *via* CPC–chitosan scaffold greatly increased the osteogenic gene expressions, ALP activity, and bone mineral synthesis by hPDLSCs. HPL concentration in CPC was directly proportional to osteogenesis, including osteogenic gene expressions, ALP activity and bone mineral synthesis. Therefore, the novel hPDLSC–CPC–chitosan–HPL construct is promising to promote the bone repair and regeneration efficacy. CPC–chitosan + 10.63% HPL scaffold increased the Runx2, OSX and Coll1 gene expressions, ALP activity and bone minerals by nearly 3 folds, compared to those at 0% HPL. Therefore, (1) CPC–chitosan is a promising carrier for HPL, (2) HPL had excellent promoting effects on hPDLSCs, and (3) the novel hPDLSC–CPC–chitosan–HPL construct has great potential for orthopedic, dental and maxillofacial regeneration applications.

## Conflicts of interest

There are no conflicts to declare.

## Supplementary Material

RA-009-C9RA08336G-s001
