# Supplementary material for: Human periodontal ligament stem cells on calcium phosphate scaffold delivering platelet lysate to enhance bone regeneration
Source: RSC Adv. 2019 Dec 13;9(70):41161–72. doi: 10.1039/c9ra08336g (PMC9076431; doi:10.1039/c9ra08336g)
Supplement: RA-009-C9RA08336G-s001 [file RA-009-C9RA08336G-s001.pdf]

Electronic Supplementary Material (ESI) for RSC Advances.
This journal is © The Royal Society of Chemistry 2019

Supplemental file

Human periodontal ligament stem cells on calcium phosphate scaffold delivering platelet lysate to enhance bone regeneration

Zeqing Zhao,[ab] Jin Liu,[bc] Michael D. Weir,[b] Ning Zhang,[a] Li Zhang,[a] Xianju Xie,[a] Charles Zhang,[b] Ke Zhang,*[a] Yuxing Bai,[a] and Hockin H. K. Xu,[bcd]

In present study, we also evaluated the cell proliferation of the CPC-chitosan+21.25% HPL group. The component of CPC-chitosan+21.25% HPL group is listed in the table below.

Group name	CPC powder (mg)	Chitosan powder (mg)	HPL (mg)	Distilled water (mg)
21.25% HPL group	75	3.75	21.25	0

Figure 1 shows representative live/dead images of hPDLSCs on CPC scaffolds at 1, 7 and 14 days, respectively. Cells of CPC-chitosan+21.25% HPL group appeared to be well attached to the CPC-chitosan scaffold.

Figure 2A shows the live cell density of all groups. Compared to control group, CPC-chitosan+21.25% HPL group had a lower live cell density ($p < 0.05$). Figure 2B shows the percentages of live cells at 7 and 14 days.

Figure 3 shows the results of the CCK-8 assay. Compared to the control group, the proliferation of CPC-chitosan+21.25% HPL group was slower ($p < 0.05$).

These results show that the 21.25% HPL group exhibited a lower cell proliferation

and viability when compared to control group. This may be due to a negative-feedback regulation by an excessive dosage of growth factors at the highest HPL dosage used in the present study.[1,2] Negative feedback plays a critical role in wiring signaling pathways, and it helps stabilize the regulatory systems of the cells.[3] Negative feedback can shut off or modulate the activation of the incoming signaling pathway to prevent inappropriate cellular response resulted from the sustaining and amplifying response to extracellular signals (such as growth factors).[4] Therefore, negative feedback is important in protecting cells from the uncontrolled growth and developmental aberrations.[5] Excessive dosages of growth factor may trigger negative feedback and impede the cell proliferation by inhibiting the related signaling pathways. These results in the present study are consistent with a previous study[2] that showed that a significant enhancement in human dental pulp stem cells (hDPSCs) proliferation was observed when using 1% and 5% HPL-containing culture media. The 5% HPL group had a higher cell proliferation rate. However, when the HPL in the culture medium was increased to 10%, a significant inhibition of cell proliferation was observed.[2] Therefore, a proper concentration of HPL could increase the cell proliferation, and this function was dose-dependent; however, an excessive HPL concentration could impede the cell proliferation.

References

1. U. L. Lee, S. H. Jeon, J. Y. Park and P. H. Choung, *Regen Med*, 2011, **6**, 67-79.

2. B. Chen, H. H. Sun, H. G. Wang, H. Kong, F. M. Chen and Q. Yu, *Biomaterials*, 2012, **33**, 5023-5035.

3. X. Yan, X. Xiong and Y. G. Chen, *Acta Biochim Biophys Sin*, 2018, **50**, 37-50.

4. N. Perrimon and A. P. McMahon, *Cell*, 1999, **97**, 13-16.

5. M. Freeman, *Nature*, 2000, **408**, 313-319.

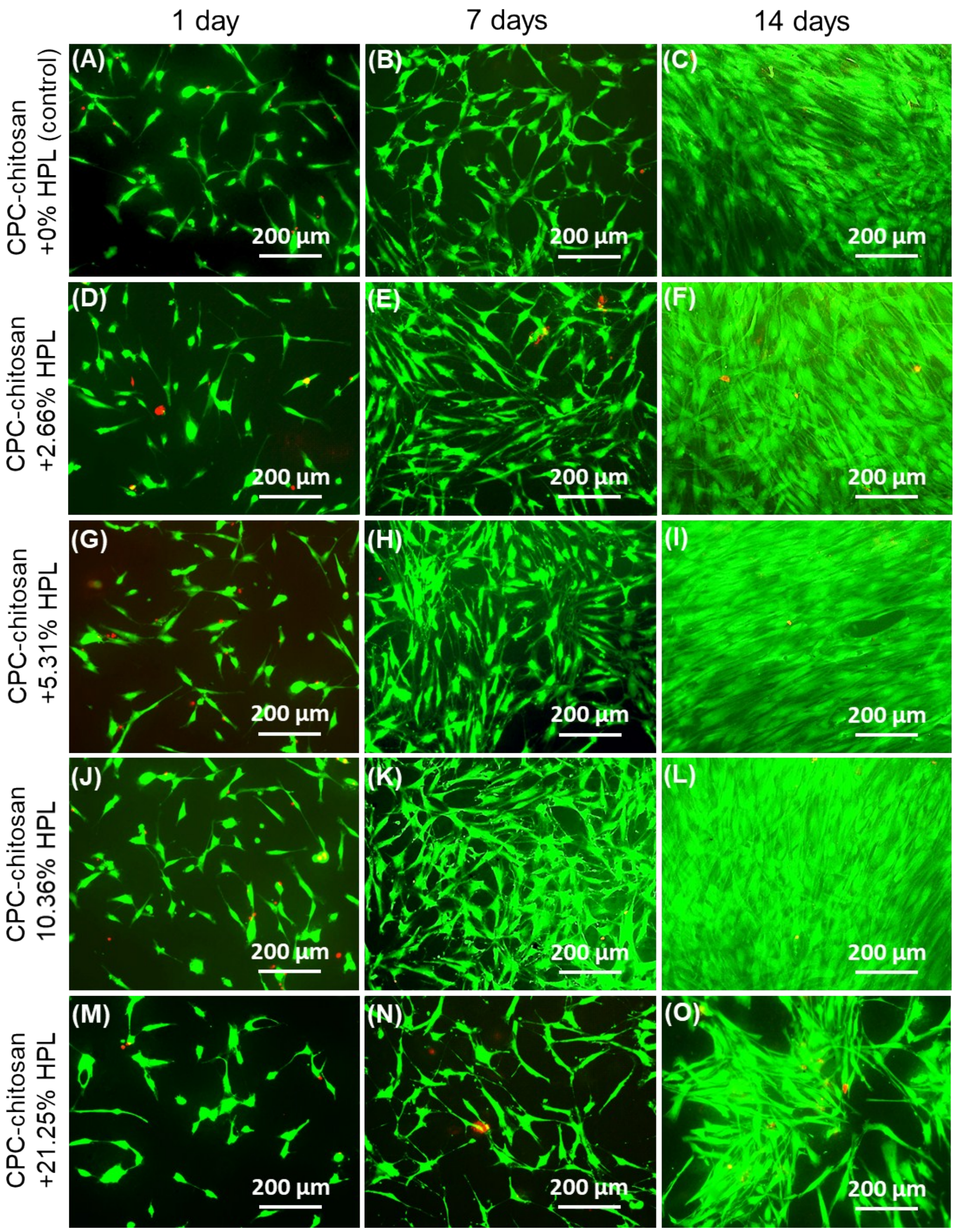

Figure 1 Representative live/dead images of hPDLSCs on CPC scaffolds for 1, 7 and 14 days for: CPC-chitosan+0% HPL (control); CPC-chitosan+2.66% HPL; CPC-chitosan+5.31% HPL; CPC-chitosan+10.63% HPL; CPC-chitosan+21.25% HPL. The number of live cells (stained green) increased with time. Live cells were numerous while the dead cells (stained red) were few. Cells of all groups appeared to be well attached to CPC scaffold.

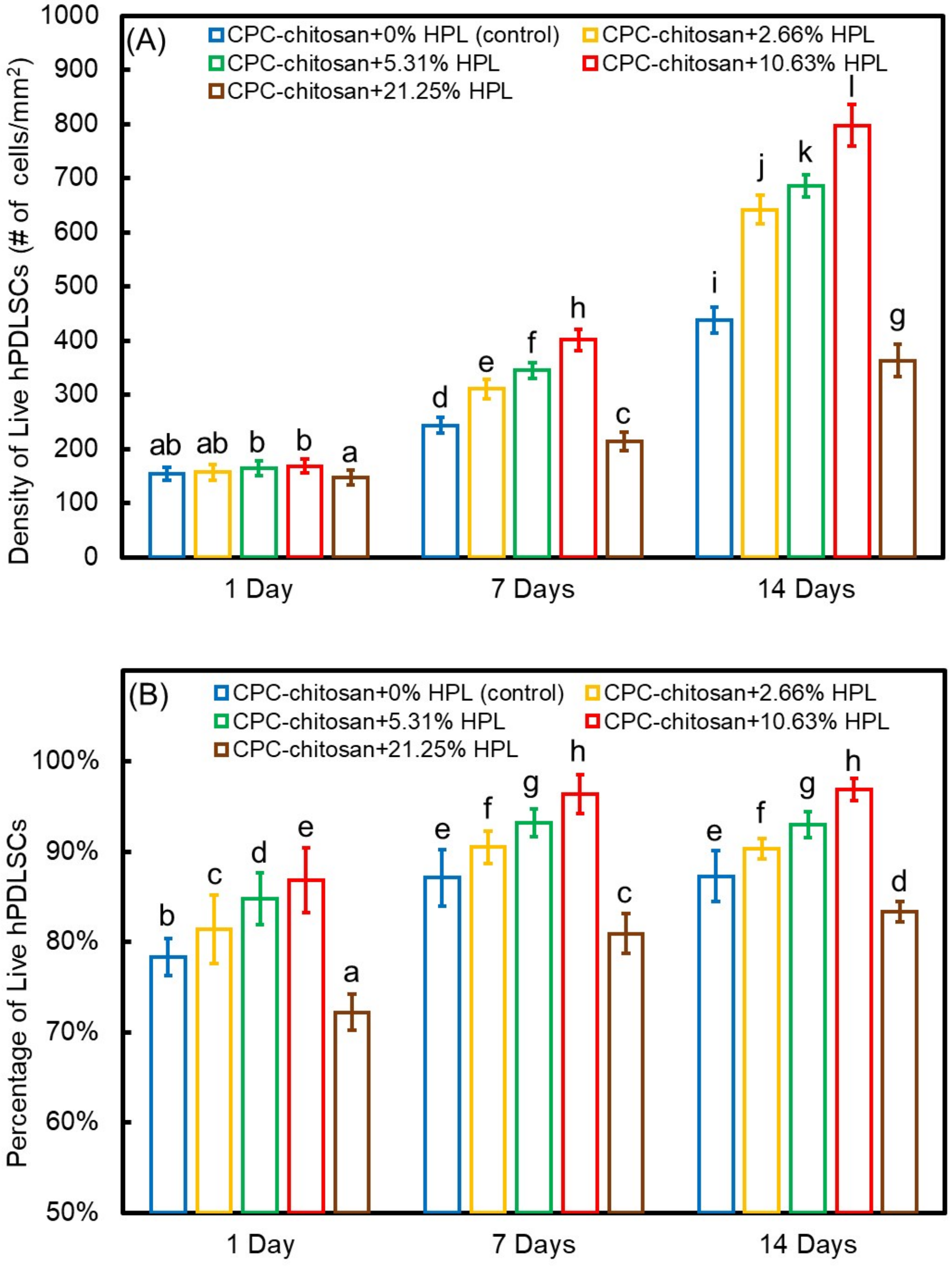

Figure 2 hPDLSC viability on CPC scaffolds (mean ± sd; n = 5). (A) The live cell density of all groups increased with time due to proliferation. (B) The percentage of live cells of all groups was about 70% to 90%. In each plot, values with dissimilar letters are significantly different from each other.

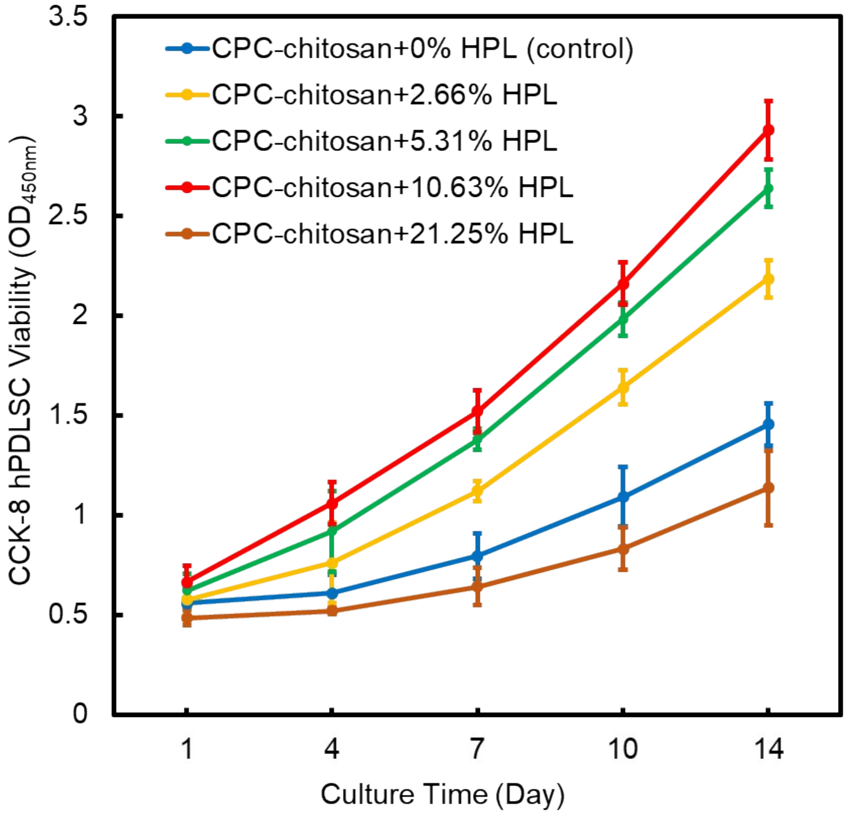

Figure 3 The hPDLSC proliferation on CPC scaffolds via CCK-8 assay (mean ± sd; n = 6). CPC-chitosan+10.63% HPL group had the greatest cell proliferation rate.
